# Reply to: No compelling evidence for early small-scale animal husbandry in Atlantic NW Europe

**DOI:** 10.1038/s41598-022-05074-5

**Published:** 2022-01-26

**Authors:** Philippe Crombé, Kim Aluwé, Mathieu Boudin, Christophe Snoeck, Liesbeth Messiaen, Dimitri Teetaert

**Affiliations:** 1 Department of Archaeology, Ghent University, Sint-Pietersnieuwstraat 35, 9000 Ghent, Belgium; 2 Gate Bvba, Dorpsstraat 73, 8450 Bredene, Belgium; 3 Royal Institute for Cultural Heritage, Jubelpark 1, 1000 Brussels, Belgium; 4 Research Unit: Analytical, Environmental & Geo-Chemistry, Department of Chemistry, Vrije Universiteit Brussel, AMGC-WE-VUB, Pleinlaan 2, 1050 Brussels, Belgium; 5 G-Time Laboratory, Université Libre de Bruxelles, ULB, 50, Avenue F.D. Roosevelt, CP 160/02, 1050 Brussels, Belgium; 6 Maritime Cultures Research Institute, Department of Art Sciences and Archaeology, Vrije Universiteit Brussel, MARI-LW-VUB, Pleinlaan 2, 1050 Brussels, Belgium

**Keywords:** Stable isotope analysis, Archaeology

**replying to**: N. Ø. Brusgaard et al.; *Scientific Reports* 10.1038/s41598-022-05073-6 (2022).

We would like to thank Brusgaard et al.^1^ for their critical reading of our paper^2^ and keeping the debate on the neolithization process of the NW European lowlands ongoing. Before addressing the comments raised by the authors, we would like to emphasize that our paper was mainly intended to present and discuss new evidence demonstrating the presence of domesticated animals well before 4300/4000 cal BC in light of the long-term debate on the pace and timing of the neolithization of the lowlands beyond the agro-pastoral frontier. Our argumentation is primarily based on two bones of sheep/goat from the site of Bazel, dated between ca. 4700 and 4500 cal BC, and we clearly stated in our paper that it is these two bones that provide the strongest evidence for early domesticated animals within the Scheldt basin, as they are clearly domestic in origin^2^. Contrary to Brusgaard et al., we believe these constitute, so far, the oldest examples of domesticated animals within a forager context and are definitely older than the dated sheep/goat bone from Hardinxveld. The latter is proven by a failed chi-test (R_Com X2-Test: df = 2 T = 8.523(5% 6.0)) when trying to calculate the average of the two dates from Bazel with the one from Hardinxveld. Similarly, the site of Bazel yielded the oldest known cereal grains, dating approximately 500 years earlier than elsewhere within the NW European plain^3^. Hence, in our view the site is exceptional as it provides the first irrefutable proof of the introduction of domesticated plants and animals beyond the agricultural frontier during the first half of the 5th millennium cal BC.

In addition we discussed in our paper some bones of *Bos* specimens which, based on osteometrics (e.g. proximal width metacarpals) might be domesticated, although we mention that (some of) these might also belong to small female aurochs. Nevertheless, we decided to further include these bones in the discussion as possible extra indication of the early introduction of domesticates at Bazel. Brusgaard et al., on the other hand, argue against the domestic nature of these animals based on an inter-site analysis of the Logarithmic Size Index. According to them, the LSI of Bazel points at the presence of mainly aurochs rather than cattle, as there is a substantial overlap with the LSI data from late Mesolithic Ertebølle sites, which contain mainly aurochs. This, however, is not surprising as the vast majority of the Bazel-bones included in their LSI analysis (cf. Brusgaard et al., supplementary data^1^) has already been attributed to aurochs in earlier studies based on osteometrics^2,4^. In these studies just 4 out of the 21 LSI-analyzed bone samples were identified as *Bos taurus*, including the two metacarpal bones which were radiocarbon dated between ca. 4800 and 4600/4500 cal BC. The fact that these four bones are situated at the lower tail of the LSI range of Bazel (LSI: −0.095/−0.136), and hence belong to species which are much smaller than the smallest Ertebølle aurochs (LSI: −0.05), clearly hints at their domesticated nature. In fact these values closely match with the LSI of cattle found within the Neolithic Linearbandkeramik and Hazendonk Cultures (cf. Brusgaard et al., Fig. 1^1^). As such, the LSI analysis conducted by Brusgaard et al. supports our statement about the likely presence of some domesticated *Bos* specimens at Bazel already during the first half of the 5th millennium cal BC. Yet it is clear that only genetic analyses can provide firm confirmation, but awaiting these we think it is important to approach the neolithization process with an open mind.

After all, it is difficult to explain why during the first half of the 5th millennium cal BC only sheep/goat would have been introduced into the lowlands, while other domesticates from the livestock of adjacent Neolithic cultures (e.g. cattle and pig) were not. We know that from 4300 cal BC onwards local livestock in the lowlands was dominated by pig and cattle, as demonstrated by the Dutch Swifterbant sites S3^5^ and Schipluiden^6^. In fact, sheep/goat seem to be virtually absent from Dutch faunal assemblages from the late 5th till the late 4th millennium cal BC^5,7^. So, why would indigenous hunter-gatherers have preferred to first obtain only sheep/goat? Similarly, among the oldest cereal grains at Bazel, no preference for a particular cereal type can be observed, as grains of both *Triticum aestivum* s.l./*turgidum* s.l., *Triticum* cf. *dicoccum*, and *Triticum* sp. were found during excavations. Furthermore, these early cereals clearly demonstrate the existence of networks involving exchange of economic commodities with adjacent farming communities from the loess area as early as 4850/4600 cal BC. Within this context the import of domesticated animals seems much more plausible. So, rather than completely ignoring the early *Bos* sp. bones at Bazel, we think it is better to include them in the discussion as potential evidence for domesticated animals.

Concerning the difference in frequency of *Bos taurus* between the faunal assemblages studied by Ervynck et al.^4^ and Crombé et al.^2^, the most likely explanation is to be found in sampling differences. Over 85% of the cattle bones within the latter assemblage, including nearly all fragments of horncore and cranium, was retrieved from a very restricted zone of about 5 to 6 m radius. This implies that most of these bones might belong to just one or a restricted number of animals.

Further in our paper^2^ we also discussed the nature of the domesticated animals found at Bazel, by exploring the possibility of small-scale local husbandry prior to 4300/4000 cal BC. Based on different, mostly indirect lines of evidence, we concluded that early husbandry might have been possible, but that there is a strong need for further analysis mainly focusing on isotopes to verify this. Brusgaard et al. contest this by claiming that our study *“does not provide new insights into the timing of incipient animal husbandry outside the loess belt.”* However, they do not discuss one of the most important arguments in favor of early stock-breeding, i.e. the indication of the use of plant winter fodder at two contemporaneous Swifterbant Culture sites, situated in the vicinity of Bazel. At Doel-sector B^8^ and sector M^9^ high numbers of mistletoe charcoal and ivy seeds were found within several surface-hearths, dated roughly between ca. 4600 and 4000 cal BC (Fig. 1). Both evergreens are known to have been used as fodder widespread over Europe from the Middle Neolithic until Medieval times to compensate for the restricted availability of grass during dry summers or snowy winters and/or over periods of stalling (^9^ and references therein). We consider this to be a strong indication for local husbandry in the Lower-Scheldt valley at least from 4600/4500 cal BC onwards, which is 200 to 300 years earlier than the currently assumed start of local husbandry in the Dutch wetlands and synchronic with the oldest sheep/goat bones from Bazel (Fig. 1). This is further corroborated by the apparent disappearance of aurochs from the faunal spectrum by the mid of the 5th millennium cal BC. At Bazel all dated aurochs bones (n = 7)^2^ are situated in the first half of the 5th millennium cal BC, the youngest one dating to between ca. 4600 and 4500 cal BC (1 sigma).

**Figure 1:**
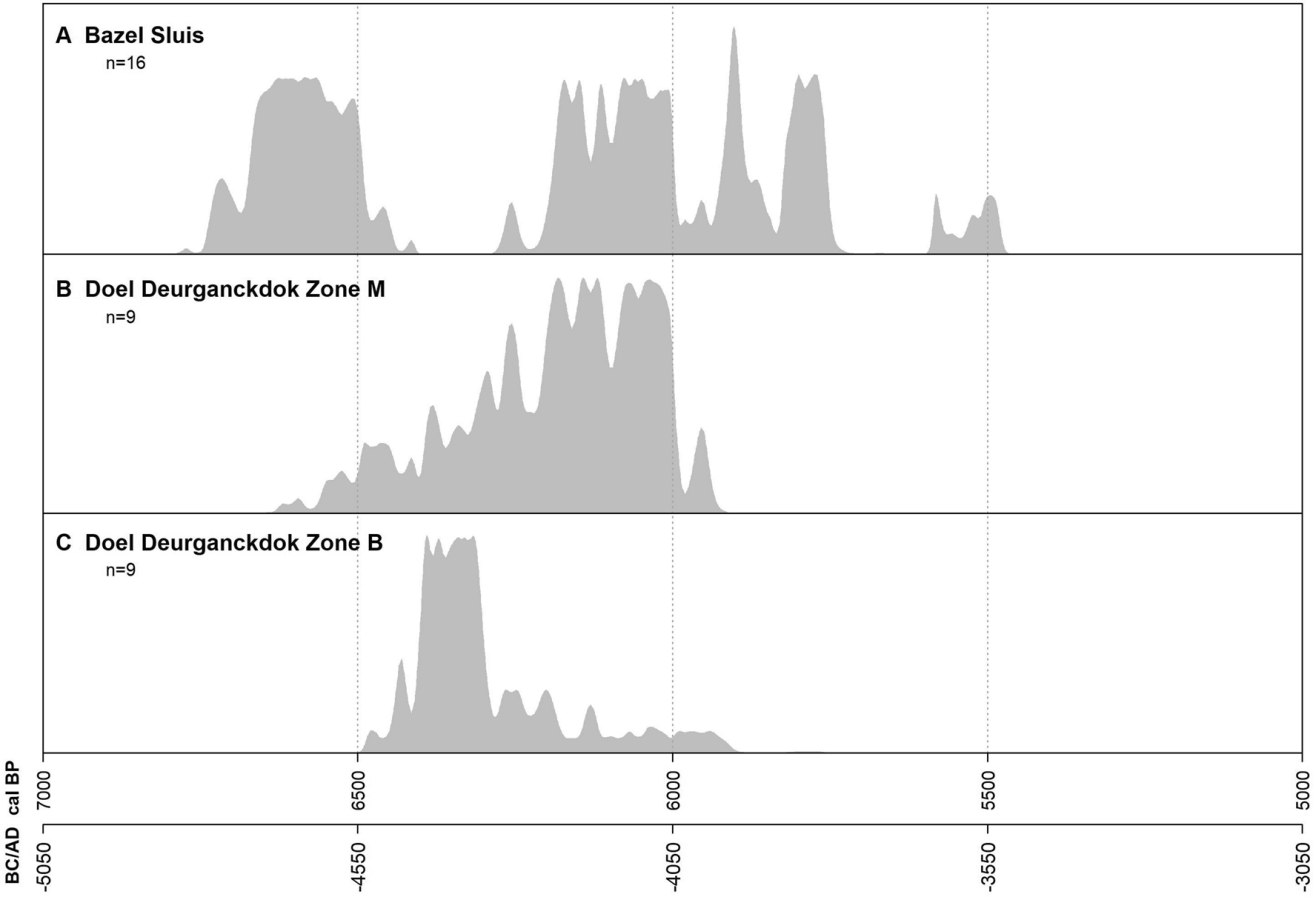
Sum probability distribution of radiocarbon dates from three Swifterbant Culture sites in the Lower-Scheldt valley. (**A**) dates on bones from sheep/goat and possible cattle; (**B**,**C**) dates on plant macroremains from surface-hearths. Both Doel-sites yielded evidence of the use of plant winter fodder (mistletoe and ivy) from ca. 4600/4500 cal BC.

Concerning the comments raised on our interpretation of the δ^13^C, δ^15^N and ^87^Sr/^86^Sr values, we fully agree that there is a need for regional carbon and strontium isoscape studies and that many factors besides environmental settings could be in play. However, waving away the observed interregional carbon differences as resulting from differences in age and size of the animals seems a bit reductive. This particularly holds for the sheep/goat remains from Bazel for which the differences in the carbon values with specimens from other sites in the loess area are too significant to be explained solely by size and/or age differences (Fig. 2). The sheep/goat bones from Bazel do not overlap at all with other sites but form a very distinct group separated by at least 1 to 2‰ from the latter. Recent statistical research^10^ has convincingly demonstrated that δ^13^C signatures vary on a latitudinal scale across Europe as a result of differences in the natural environmental parameters of the local habitats. At Bazel this is supported by the close correspondence of the carbon signal between sheep/goat and wild herbivores, both aurochs and red deer, which most likely reflects feeding on plants from similar ecological niches (Crombé et al., Fig. 6^2^). Either this is related to the seasonal uptake of ^13^C-depleted winter fodder (cf. above)^11^ or to domesticated animals browsing in the local forests. Either way, it renders additional indirect support for local husbandry, although in the latter case the presence of feral animals, which escaped from farmer settlements at distances of  ca. 80 km from Bazel needs also to be considered^12^. Anyhow, the current isotope evidence seems not really in support of gift-exchange of butchered animal parts, as proposed by Brusgaard et al.

**Figure 2:**
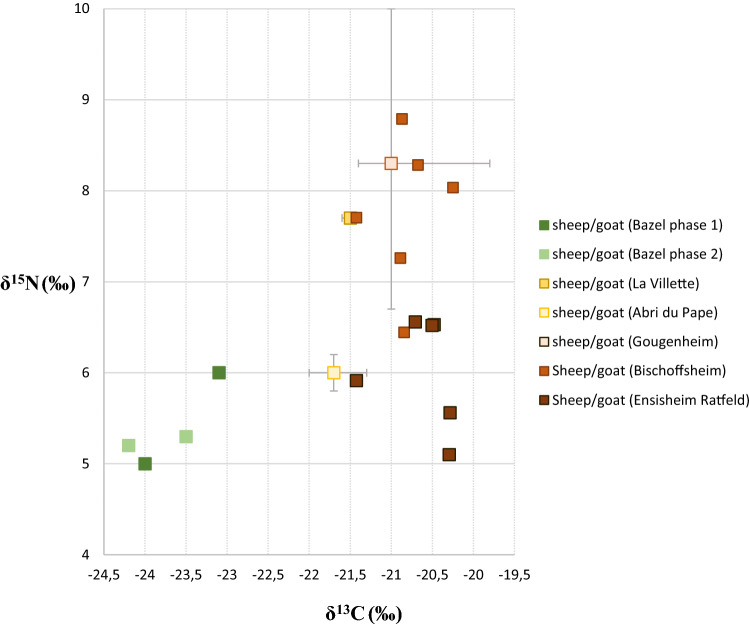
Stable isotope data obtained on sheep/goat bones from the site of Bazel, compared to data from early and middle Neolithic sites in the adjacent loess region of Belgium and France^10,15^.

Nonetheless, we are fully aware that strontium isotopes are much better suited for tracing the origin of the first domesticates. Unfortunately so far strontium data is only available for the youngest cluster of cattle remains at Bazel. We agree that strontium values of at least three samples from Bazel also fit with the signatures of the southern Netherlands, in particular from the dry coversand area south of the Meuse/Rhine (isoscapes C & D^13^). However, it is very unlikely that cattle found at Bazel originates from that area since currently there is hardly any evidence of 5th millennium farming communities there^14^. So, we believe that our initial careful suggestion that either these cattle originate from different areas, possibly within Belgium but other places are also possible, or they were all grown locally but fed in different environments, still stands.

In conclusion, contrary to Brusgaard et al. we believe that our study does change the existent image of the NW European lowlands prior to 4300/4000 cal BC by adding direct evidence of domesticates, in particular sheep/goat and possibly some cattle, as well as indirect evidence of early small-scale husbandry, mainly based on the remains of winter fodder at Doel, at least from ca. 4600/4500 cal BC. Lacking evidence of the use of winter fodder prior to this date, the nature of the older specimen of potentially domesticated animals dated ca. 4800–4600 cal BC at Bazel currently remains unclear. According to the stable isotopes these might equally well belong to feral animals shot by local hunter-gatherers. Clearly Bazel, situated at relatively short distance beyond the agro-pastoral frontier of the European loess region, is an important site for understanding the start of local husbandry in the NW European lowlands, as further in-depth analyses of bone isotopes and charred plant/wood remains from this site would certainly yield interesting new data.

## Data Availability

No datasets were generated or analyzed during the current study, others than those published in Crombé et al.^2^, Meylemans et al.^3^, Ervynck et al.^4^, Deforce et al.^8,9^, Goude & Fontugne^10^ and Bickle^15^.
